# Supplementary material for: Value-complexity tradeoff explains mouse navigational learning
Source: PLoS Comput Biol. 2020 Dec 11;16(12):e1008497. doi: 10.1371/journal.pcbi.1008497 (PMC7758052; doi:10.1371/journal.pcbi.1008497)
Supplement: Attachment — Submitted filename: Review.pdf [file pcbi.1008497.s001.pdf]

Review of the manuscript "Value-complexity tradeoff explains mouse navigational learning" by Amir et al.

The authors present a normative approach to the problems of mice navigating in a Morris water maze and the learning displayed in behavior. The theory describes the motion of a mouse as resulting from a control force applied to an otherwise overdamped random walk in a well. The control history is obtained by trading off a value function (which quantifies how rapidly the mouse reaches the platform and how close to it the mouse swims otherwise) with a complexity cost (which quantifies how different the controlled trajectory is from the free random walk). The authors then fit this model to both wild-type (WT) mice and mice that present a mutation on the Pogz gene (HET). This enables them to study the behavior of mice directly (e.g., by quantifying how the latency of swims evolves during learning) as well as through the lens of the model, by quantifying the inferred value and complexity cost associated by WT and HET, male and female, mice, at different stages of learning. In the author's approach, learning is viewed as a modulation of the parameter that governs the tradeoff between value and complexity cost; over time, it varies such as to allow for a larger value, at the expense of a larger computational cost. In this connection, an intriguing result is that WT mice present a non-monotonic evolution of the complexity cost during learning, while HET do not. The authors interpret this as instantiating a two-stage learning, in which WT mice first increase their value at the expense of complexity, and later simplify their control algorithm, in analogy with a similar phenomenon that some of the same authors have found in the dynamics of learning in deep networks.

This is a wonderful paper, which I greatly enjoyed reading. I fear that other reviewers or readers may focus on the experimental relevance of the approach and on whether it is well suited to such questions as differentiating WT from HET mice. One can certainly quibble about such questions, but I don't think that it would be the adequate vantage point from which to either evaluate or learn from this work. In my view, the manuscript has the high merit of introducing several innovative and interesting ideas, which are thought-provoking and can in turn stimulate further worthwhile research. Developing normative theories of behavior and constructing useful quantifications of 'complexity of control' are extremely important endeavors, with broad applications from ecology to cognitive science to robotics. Even though the approach developed here is related to classic work (Gaussian-linear control theory, Kalman filtering, etc), the authors put forth interesting proposals on both aspects, which are intellectually stimulating; if only for this reason, this paper should definitely be published. Too often papers are evaluated on whether they can reproduce a specific data set, and too rare are the published papers that contain interesting, new ideas.

I mention also that the Discussion presented in the paper is a pleasure to read and raises a number of interesting points as well as questions for future research. I am looking forward, in particular, to the authors' future work combining the present approach with position inference carried out by the mouse.

I will comment on the paper from the vantage point just mentioned, which emphasizes the intrinsic interest of the theoretical contribution rather than the predictive or explanatory power of the model developed. From this point of view, I do not have serious criticisms. However, I suggest hereafter that a number of aspects of the approach be discussed further or clarified, so as to help the reader understand the theoretical construct and consider the implications of the model.

Major points:

1. In the way the theory is introduced, there is some blur between cognitive and mechanical aspects. Naïve mice are described as overdamped random walkers in a well; so naïve mice do not come with an explicit objective function, which could then couple with the presence of a platform. In principle, one could think of describing the behavior of a naïve mouse through some objective function, which would presumable depend on whether the mouse 'knows' that there is a platform. The authors could maybe describe somewhat more in detail what is meant by a 'naïve mouse' and why the random walk description is adequate. Incidentally, also, the authors mention that naïve mice mostly swim along the wall of the circular tank, a description at odds with that of a random walk.

2. It would be helpful to have a more detailed discussion both of the choice of the form of the complexity cost and of its possible relation with cognitive and metabolic costs, especially as this is such a central contribution of the paper:
    a. The authors mention large deviation theory and Sanov's theorem in passing; a more substantiated description, here, would be welcome.
    b. As the authors describe, the complexity cost quantifies how far away from free behavior the control variable forces the trajectory (or, more precisely, how unlikely such a deviation is). Even more basically, it depends on properties of the control, such as its magnitude. Should we interpret this as a proxy for a cost of muscle activation or some such metabolic cost?
    c. The authors also indicate that their complexity cost can be associated to a form of complexity of cognitive processing. It would be nice to see a more thorough discussion of this connection, too. For example, if the control forces the mouse to progress in a straight line forward, this will incur a high complexity cost, but it is not clear why this should be cognitively costly (even if it may be costly in terms of muscle control).

3. The authors propose to solve for the control variable by minimizing a functional that trades off value and complexity cost. I have two questions in connection with this proposal:
    a. Since the problem is stochastic, should it not be solved instead by propagating the effect of the control variable into the future, i.e., by solving a Bellman equation along the trajectory?
    b. How are the boundary conditions imposed? In relation to this question, also, it wasn't clear to me how the authors treat the duration of each trial. They mention that the trial ends either when the mouse reaches the platform or when the experimenter places the mouse on the platform (if the mouse hasn't found it before some threshold latency). To what extent does the choice of latency threshold influence the results? (I suppose that longer trials would tilt the balance toward value and away from complexity, if the mouse spends some time swimming around the platform before mounting on it, as the data suggests.)

4. The form of the modified optimal control variable, Eq. (19), which includes the effects of the complexity cost, is a key result of the paper. The authors should present a full derivation of this result in the appendix. Furthermore, currently the corresponding section in the appendix

starts by providing the solution, namely the Riccati equation in continuous form. I understand that the derivation is more direct in the continuous case, but the continuous theory is not presented in the main text, since the complexity cost in defined for discretized time. It would be advantageous to have both continuous and discretized models laid out carefully, especially if the continuous Riccati equation is used.

5. Figure 4 and its discussion were confusing to me. It is unclear why this specific analysis and representation were opted for, and what one should conclude from them. Also, I suspect that the figure could be improved.

6. The authors refer to learning in a somewhat unusual way. Here, learning corresponds to variation in the parameter, β, which quantifies the relative importance of value vs. complexity cost. In successive days, the value of β changes in such a way as to allow for higher value, at the expense of higher complexity cost. But this change in β does not result from the acquisition of information (at least not explicitly so, in the model), as one would expect in connection with actual learning. Could the authors discuss this view of learning in greater detail? For example, once the mouse 'knows' that a platform exists in the tank, i.e., by the second day or a later trial in the first day, why does it not change the value of its β directly to the value it has in later days?

7. A few questions and comments pertaining to Fig. 5:
   a. I found it quite impressive that many of the data points fall so close to the theoretical bound. Clearly, the model is far from realistic, and because of its several idealized aspects (some of which the authors comment upon in the Discussion), there is no obvious reason to assume that this obtains. Can the authors comment on this observation?
   b. Both value and the complexity cost have to be evaluated from their theoretical expressions and, hence, using the parameters estimated from data. These are estimated by assuming that the behavior is optimal (i.e., minimizes the free energy). Figure 5, however, shows that behavior can lie away from the theoretical curve. Is there a contradiction in the approach, here?
   c. The grey line that exhibits mean values over mice/trials is presumably obtained from an average over all mice/trials; it displays a non-monotonic evolution of the complexity cost. Since later on, using Fig. 6, the point is made that WT mice show non-monotonicity, but HET mice do not, I wondered why the authors did not distinguish between the two groups also in Fig. 5, which would then have exhibited two different 'average curves'.

8. The authors discuss some of the connections of their work with past work, including on control theory and inference. It may be nice to mention also connections with rate-distortion theory and the information bottleneck.

<u>Minor points:</u>

9. I suspect that the authors decided to describe the dynamics in a Hamiltonian formalism rather than in a Lagrangian formalism because it yields a Markov property useful, e.g., for

discretizing time and for calculating the likelihood of a trajectory in data fitting. It would be good to explain this.

10. Equation (3): in upper right block of the matrix, should the elements not be “1/*m*” instead of “1”?

11. Equation (4):
    a. What is the rationale for assuming noise with fixed variance? In principle, a control variable could also be applied to modulate the magnitude of the noise.
    b. What is the rationale for assuming the presence of noise in both position and momentum?

12. I suggest using square brackets, rather than round brackets, around the argument of functionals (e.g., in Eqs. (7) and (14)), as this notation is more common and is also helpful to distinguish functionals from functions.

13. In Eq. (7), the parentheses are missing around the integrand.
