# Supplementary material for: Value-complexity tradeoff explains mouse navigational learning
Source: PLoS Comput Biol. 2020 Dec 11;16(12):e1008497. doi: 10.1371/journal.pcbi.1008497 (PMC7758052; doi:10.1371/journal.pcbi.1008497)
Supplement: Attachment — Submitted filename: MWM response letter.pdf [file pcbi.1008497.s002.pdf]

“Value-complexity tradeoff explains mouse navigational learning” - response letter

Response to reviewer #1

The manuscript describes a formal mathematical framework intended to capture the key features of learning to navigate a water maze by rodents, and applies it to model or fit behavioral data from wild-type and Pogz-gene-mutated mice. There are several components to the theoretical work reported here: (a) formulating a simplified model of the behavior of naïve animals, as a damped oscillator; (b) defining a cost function that combines the notion of favoring trajectories that lead to the submerged platform with the notion of adopting as simple a strategy as possible; (c) fitting the model to the data and extracting parameters that succinctly describe the learning process, in particular the inverse temperature Beta.

I admire the ambitious attempt to theorize about a behavior that many labs across the world have observed endless times, and for which there seems to be a need for a comprehensive theory. The theory developed here is undoubtedly elegant and mathematically well formulated, and it is abstract enough to generate that sense of mystical awe that is a necessary attribute of a psychologically satisfying theory. Personally, however, I am a bit skeptical about the implicit “behaviorist” assumption that either naïve rodents, with all their complexity and individual differences, or the learning process they demonstrate in the water maze, with its interaction with specific psychological factors, can be reduced to the basic physics of a harmonic oscillator or to the basic informatics of Kullback-Leibler minimization. If such a reduction were indeed possible, it would suggest that mice (at least when they learn to navigate a water maze) do not really need their brain, a standard algorithm is sufficient – which was after all the spirit in which behaviorism proceeded in the 50’s, before being replaced by cognitive neuroscience. Looking at data like that in Fig.5, with its wide scatter of individual data points over orders of magnitude, does not help quell my skepticism. Still, simplified models can be very useful even if they neglect important factors and only approximately fit the data, so I read the manuscript with this consideration in mind.

Thank you for your insightful comments. We understand the skepticism regarding the attempt to reduce complex and individually variable animal behavior to simplistic mathematical models. However, this is not the main objective of the model. Rather, it is to demonstrate a novel framework for quantification of biological learning in general, based on the tradeoff between performance and behavioral complexity. The finding that even an extremely simplified model, implementing this idea, provides interesting and novel insights on a well studied experimental paradigm, illustrates the usefulness of this novel perspective for quantifying learning.

Specific responses

Component (a), the linear oscillator model of naïve trajectories in a water maze, is striking in its simplicity and fun to read about. I was underwhelmed, however, by the limited discussion of its suitability for the problem at hand. Yes, they say that a linear model is required by the formalism to be adopted, and that it is not intended to model individual trajectories, of course; but I would have expected to see more of a discussion of what the model misses out, what are the major collective and individual avenues of departure from the model, and to what extent one could imagine to account for them in an extended description, perhaps as a perturbation, to stay in the physics domain.

We expanded the discussion of the Naive mice model, its strengths and limitations. In the Summary section of the discussion we write:

> *The tendency of naive mice to swim in wide, quasi-circular arcs near the wall of the tank led us to model their trajectories with the dynamics of a stochastic, damped oscillator. Naive model trajectories were shaped by a balance between the tendency of the noise-free trajectories to converge spirally towards the center of the pool on the one hand, and the noise that drives the trajectories outwards on the other hand. This simple model captured properties of naive mice motion surprisingly well.*

There is no doubt that this is not what mice are doing, and we discuss this further in the next section of the discussion:

> *For example, a more realistic description of swimming trajectories could be obtained by using non-linear dynamics or a different noise model, e.g., multiplicative, control-dependent noise which has been proposed as more suitable for the description of sensorimotor behavior than additive, control-independent one … One disadvantage of these approaches is that they may require the introduction of artificial devices to reproduce the tendency of mice to swim in circular arcs close to the walls on their initial exposure.*

In the balance between a simple model of essential features, and a detailed model that attempts to capture all complexity, our model is certainly biased towards simplicity. Nevertheless, the model captured crucial features of real experimental data, and at the end, this is its best justification.

Component (b) is the core contribution of this study, and is summarized by the attractive notion that learning (in this very specific instance) proceeds by maximizing value for the learner while trying to minimize or at least to control the increase in its (behavioral?) complexity, where complexity is defined in a somewhat recursive fashion not based on intrinsic parameters of the behavior, but rather on how behavior departs, microscopically, from that of a naïve agent, who has not learnt a thing. The application to the data then leads to the stimulating suggestion that complexity might increase early in learning, and decrease in its final stages. To me, this is another under-discussed aspect of the study.

We address, in order, the specific questions raised by the reviewer:

1. Is it a strong result?

We added to the discussion the result of a statistical test (Kolmogorv-Smirnov) showing a significant difference between the distributions of trajectory complexities on day 2 (mean complexity = 2.35) and day 4 (mean complexity = 1.62) for female wild-type mice.

2. What does it really hinge on?

This is an insightful and extremely important question - the reduction in complexity may reflect some obvious parameter of the swimming paths. We therefore looked in some detail for features of the swimming paths that were correlated with the reduction in complexity, but we couldn't find any single feature that could account fully for the behavior of complexity.
We added to the paper a report on a negative correlation between complexity and mean swimming velocity for female wild type mice, suggesting that the reduction in complexity is related to a reduced swimming velocity. This is in line with our finding that a tendency for flotation behavior can explain the lower complexity of mutant vs wildtype mice (Fig. 7). However, neither of these fully accounts for the differences between the groups. Indeed, our model predicted a nonmonotonic profile in velocity as well as complexity. In spite of the correlation we observed between complexity and velocity, we were not able to detect the non-monotonicity of the velocity in the data. Thus, complexity could be a more sensitive measure of behavior than purely velocity based indices.

3. Would more standard measures of trajectory complexity also produce it?

We don't know - complexity has not been defined in any paper we know of that analyzes swimming paths in the MWM. As far as we know this is the first model of the water maze providing a measure of complexity at the level of single trajectories. Also, the notion of complexity as we define it is different from the naive notion of complexity - as we write towards the end of the introduction,

> *Indeed, while the directed swimming trajectories of the trained mice are simpler than the winding swimming trajectories of the naive ones, they require moment by moment application of precise motor behaviors which are unlikely to result from the unconstrained behavior generating the winding trajectories of naive mice. Thus, generating direct trajectories requires more computational effort than generating meandering ones, on top of any differences in terms of metabolic or motivational costs. This increased computational effort is precisely what our complexity measure attempts to quantify.*

4. How does it square with the discrepancy between model-based prediction and observation in the case of speed (Fig.3c)?

The model failure to detect the predicted non-monotonic velocity profile may be due to the large variance in velocity data - note how large are the error bars for the velocity in Fig. 3c relative to those in Figs. 3a,b and d. It could also indicate the existence of other low-level properties of late trajectories accounting for their reduced complexity, although we were unable to robustly characterize such features. Because of the model's simplicity, we are more surprised by its successes than by its failures.

5. Is it reflected at all in the monotonic trends in Fig.3a,b,d, or if not would those trends have resulted from basic value maximization, or perhaps assuming a more basic constraint that the complexity as defined here?

The quantities presented in these panels are all related directly to the value. These trends are therefore related to value maximization.

Component (c) is a key component of the study, which distinguishes it from too abstract an academic exercise, and I much appreciate it. I would have liked, however, to see more of a discussion of the specifics of the genetically modified mice used, and of what the analysis might reveal about them, also in relation to gender effects.

The Methods section now contains a more detailed description of the genetic manipulation. We also added a paragraph at the end of the discussion section, relating our findings to abnormal behavior in humans with mutations in POGZ.

I would like to see more trajectories, produced by mice and by the model.

We redesigned the presentation of empirical and model generated trajectories (Fig. 2) which now compares empirical and simulated trajectories for naive and trained mice in a 2x2 panel with 4 trajectories in each panel.

In conclusion, I am impressed by the mathematical sophistication and technical prowess of this study, and I would enjoy a deeper analysis of its implications for theoretical neuroscience.

We thank the reviewer for this assessment of our work. Given the length of the current paper, we believe that deeper analysis of its implications should be kept for future work.

Response to reviewer #2

This paper describes a new normative model which quantifies rodent trajectories during the well-established spatial memory task of the Morris water maze. This approach uses dynamical systems theory from statistical physics. The authors compared predictions from the model with experimental data from wild type and transgenic mice lines (N=51).

The key contribution is observed trajectory data can be explained in a trade-off between two model-derived quantities. The value of a trajectory and the complexity of a trajectory. The value is the energetic cost and was found to monotonically increase throughout the four training days. The complexity is the likelihood of the trajectory of a naïve animal behaving stochastically. The complexity was first observed to increase as paths become more random, and then decrease as paths become simplistic. This model may be useful to researchers who are interested in understanding differences in performance across rodent types and experimental manipulations within and beyond the Morris' water maze, and more broadly for trajectory analysis in many species. Thus this work provides a potentially useful advance.
We thank the reviewer for this positive evaluation.

Below are a list of suggestions for improvements for the manuscript:
1. A key weakness with the current manuscript is the absence of another model-fit to the data for comparison to the one proposed. Currently, the model shows interesting findings such as the increase in value of time and non-monotonic relationship to complexity. However, there is no comparison to alternative plausible models.
We mention many previous models for learning in the water-maze in the discussion section. This comparison is now expanded (first paragraph of subsection 'Is the model novel?" in the discussion).
The main problem we faced is that our model sets out to explain the data at a very different level than previous models. Previous models focus on explaining spatial map formation in terms of hippocampal place cell connectivity or activation features whereas our model focuses on motor and information-theoretic constraints that are derived directly from the swimming paths. We do not assume any neuronal implementation. Thus, we believe that there is no alternative model that is comparable to ours - trying to account for the learning using a single parameter - β. We added to the discussion an explanation of these differences and why they make such comparisons difficult to conceive.

2. The authors state: "Mean velocity had the correct range of values, although it failed to show the predicted non-monotonic dependence on the trade-off parameter β (potentially because of the high variability of the experimental data).". This point should be discussed more in the discussion. What might underlie the high variability in the data not captured in the model? Why would the variability specifically affect the predicted dependence? This isn't a given and would be worth explaining more for readers.
We added to the discussion one possible source of variability in the velocity data, namely the fact that it is obtained via numerical differentiation of the position measurements. While this variability in itself should not specifically affect the predicted non-monotonicity, it would make it harder to detect using the data at our disposal.

3. There is another recent addition to reinforcement learning that also references the Todorov PNAS paper called linear reinforcement learning. It also uses a quantity derived from the cost of a trajectory similar to the value described here and is analytically solvable. This work is currently on bioRxiv and not published as of yet (https://www.biorxiv.org/content/10.1101/856849v1.full.pdf). It would be important for the authors to comment on this and consider it in their discussion.
Thank you for pointing out this interesting work. We added it to our discussion of similarities and differences between our approach and that of others such as Todorov's KL-based linearly solvable MDPs.

4. Please make clear if you are aiming to publish your code along with the publication.
Yes.

5. Figure 4 is worth re-working for greater clarity. The image shows a blurring of the arrow head with the colour scale for the arrow for the data, is this a range of arrows superimposed or information about the precision of the arrow? I would also be improved by providing a second example at a different point in the learning process and marking S for the release point to match the legend in the images.
We added boxed insets for explaining the color range of the arrows as indicating the predictions of models with different β values and showing the precision of the predicted velocity arrow. We also added the marking S for the release point and a more detailed description of the figure in the text. We found that providing a second example from a different release point created visual clutter but we hope that the changes now make for a clearer figure.

<u>Response to reviewer #3</u>

Major points

1. In the way the theory is introduced, there is some blur between cognitive and mechanical aspects. Naïve mice are described as overdamped random walkers in a well; so naïve mice do not come with an explicit objective function, which could then couple with the presence of a platform. In principle, one could think of describing the behavior of a naïve mouse through some objective function, which would presumable depend on whether the mouse 'knows' that there is a platform. The authors could maybe describe somewhat more in detail what is meant by a 'naïve mouse' and why the random walk description is adequate. Incidentally, also, the authors mention that naïve mice mostly swim along the wall of the circular tank, a description at odds with that of a random walk.

We added an explanation of what we mean by “naive mouse”, namely, a mouse that has not yet been exposed to the location of the platform. All mice are therefore naive on their first trial. As reported in the Methods, if the mouse does find the platform on its own by the end of the trial (i.e., after 60sec), it is placed on it by the experimenter. Thus, mice are no longer naive after the first trial. Since naive mice tend to swim in wide, circular arcs around the center of the tank (near the walls), we decided to model their behavior using a damped harmonic oscillator model. This is a biased random walk, or more precisely, a Langevin, or drift-diffusion dynamics. The damping factor represents drag forces due to the water and the harmonic force generates the tendency for wider arcing motion around the center. The noise accounts for randomness in motion, and prevents the trajectories from converging to the center by balancing the damping induced energy dissipation (according to the fluctuation-dissipation theorem), keeping the model mice closer to the tank walls. We used the model because it was the simplest model that could produce the circular swimming paths. We were actually surprised to see how well the model, in spite of its simplicity, reproduced observed data.

2. It would be helpful to have a more detailed discussion both of the choice of the form of the complexity cost and of its possible relation with cognitive and metabolic costs, especially as this is such a central contribution of the paper:

a. The authors mention large deviation theory and Sanov’s theorem in passing; a more substantiated description, here, would be welcome.

We added some information at the introduction. This is a general comment about the Dkl as a measure of statistical distance - it bounds the likelihood of data selected by one probability to ‘look like’ data coming from a different one.

b. As the authors describe, the complexity cost quantifies how far away from free behavior the control variable forces the trajectory (or, more precisely, how unlikely such a deviation is). Even more basically, it depends on properties of the control, such as its magnitude. Should we interpret this as a proxy for a cost of muscle activation or some such metabolic cost?

c. The authors also indicate that their complexity cost can be associated to a form of complexity of cognitive processing. It would be nice to see a more thorough discussion of this connection, too. For example, if the control forces the mouse to progress in a straight line forward, this will incur a high complexity cost, but it is not clear why this should be cognitively costly (even if it may be costly in terms of muscle control).

We added a more substantiated description of the complexity cost and its interpretation. Briefly, our complexity measure is taken as a fundamental quantifier of the computational cost involved in action generation and selection. It is not meant to serve as a proxy for muscle activation or some such metabolic cost, these are supposed to be indexed by the value functional. Swimming in a straight line towards a target, to take one example, would incur high complexity cost because in the context of the model, that includes damping and noise, it requires moment-by-moment

generation of precise motor commands to counteract damping and correct precisely for the noise. This would presumably result in higher computational and cognitive load which is what our complexity measure aims to quantify.

3. The authors propose to solve for the control variable by minimizing a functional that trades off value and complexity cost. I have two questions in connection with this proposal:

a. Since the problem is stochastic, should it not be solved instead by propagating the effect of the control variable into the future, i.e., by solving a Bellman equation along the trajectory?

Indeed, the modified Ricatti equation (Eq. 58) is obtained as the solution to a Bellman equation, incorporating both value and complexity costs, along the trajectory. Note that the fact that the problem is stochastic does not change the underlying form of the equation but rather modifies the expression for the control weight matrix R (Eq. 49) due to the effect of the noise covariance on the complexity cost (Eq. 48).

b. How are the boundary conditions imposed? In relation to this question, also, it wasn't clear to me how the authors treat the duration of each trial. They mention that the trial ends either when the mouse reaches the platform or when the experimenter places the mouse on the platform (if the mouse hasn't found it before some threshold latency). To what extent does the choice of latency threshold influence the results? (I suppose that longer trials would tilt the balance toward value and away from complexity, if the mouse spends some time swimming around the platform before mounting on it, as the data suggests.)

There are two kinds of boundary conditions which could be relevant in this context: spatial and temporal. The first, representing the walls of the tank, can be imposed using boundary conditions of the third kind (so-called Robin conditions) on the probability distribution function satisfying the Fokker-Planck or (Kolomogorov) forward equation corresponding to the naive drift-diffusion process (Eq. 1). The second, representing the finite length of trials, could be imposed by solving the finite horizon, or differential Ricatti equation (Eq. 53) rather than the infinite horizon, or algebraic Ricatti equation (Eq. 52).

We decided not to impose these conditions in our models, each for a different reason. The first, spatial boundary condition, would introduce a strong non-linear component to the model making it intractable. We preferred a simple, tractable model that nevertheless captures the essential features of the naive motion, namely, large quasi-circular arcs around the center of the tank. The second, temporal boundary condition, would add a transient, time dependent component, to the solution of the optimal control problem. These transients however converge exponentially to zero, much earlier than the latency limit of 60 s. Thus, they did not have a large effect on the solution of the control problem and we decided to neglect them. These considerations are now also mentioned in the paper. Note that the value and complexity of the empirical trajectories shown in Fig. 5 are normalized by the total swimming duration.

4. The form of the modified optimal control variable, Eq. (19), which includes the effects of the complexity cost, is a key result of the paper. The authors should present a full derivation of this result in the appendix. Furthermore, currently the corresponding section in the appendix starts by providing the solution, namely the Riccati equation in continuous form. I understand that the derivation is more direct in the continuous case, but the continuous theory is not presented in the main text, since the complexity cost in defined for discretized time. It would be advantageous to have both continuous and discretized models laid out carefully, especially if the continuous Riccati equation is used.

We changed the order of presentation of the corresponding sections in the appendix so that the solution of the Riccati equation is now presented after the reduction of the modified optimal control problem (including the complexity cost) to the form of a standard Linear Quadratic Regulator problem with a control cost term modified by the noise covariance matrix (Eq. 57). Both continuous and discrete forms of the Riccati equation are presented in the latter section.

Concerning the continuous time definition of complexity, we added the following comment to the results section after defining the discrete time complexity functional,

> *While it is possible to provide a continuous time version of complexity, at least in the Gaussian noise case, it is simpler and more transparent in the discrete case which is anyway what we computed on the empirical data*

5. Figure 4 and its discussion were confusing to me. It is unclear why this specific analysis and representation were opted for, and what one should conclude from them. Also, I suspect that the figure could be improved.

We improved the figure (the inset squares were omitted from the previous submission due to a technical error) and its description in the text. The goal of this analysis is to compare the actual velocity vector at several points along a representative empirical trajectory with the predicted velocity vectors for models with different β values, i.e. a different tradeoff between complexity and control. The figure also shows that the empirical trajectory lies within a range smaller than one standard deviation from the model predicted ones. The variance in the model predicted velocity is due to the Gaussian noise term in the model.

6. The authors refer to learning in a somewhat unusual way. Here, learning corresponds to variation in the parameter, β, which quantifies the relative importance of value vs. complexity cost. In successive days, the value of β changes in such a way as to allow for higher value, at the expense of higher complexity cost. But this change in β does not result from the acquisition of information (at least not explicitly so, in the model), as one would expect in connection with actual learning. Could the authors discuss this view of learning in greater detail? For example, once the mouse ‘knows’ that a platform exists in the tank, i.e., by the second day or a later trial in the first day, why does it not change the value of its β directly to the value it has in later days?

Our parameter estimation procedure is such that the performance at the last day of training is that of the optimal controller. Our empirical observations imply that whatever is the learning process, it is well-captured by the relaxation of the information constraint. Thus, a single 'macroscopic' variable, β, captures the 'microscopic' aspects of the learning process. Higher β can be obtained by a number of different mechanisms - improved memory of the spatial landmarks, more precise map of the environment, more precise computation of the motor commands, or better estimates of the parameters of the control task. The common denominator of all of these is the better coupling between sensory and motor processes in the brain, producing a more complex behavior in our terms. This improved coupling can be quantified by information flow from the sensory systems that identify mouse location to the motor systems that produce the movement. This is now touched upon in the discussion (last paragraph of the Summary subsection).

7. A few questions and comments pertaining to Fig. 5:

a. I found it quite impressive that many of the data points fall so close to the theoretical bound. Clearly, the model is far from realistic, and because of its several idealized aspects (some of which the authors comment upon in the Discussion), there is no obvious reason to assume that this obtains. Can the authors comment on this observation?

We added a comment on the fact that many data points fall close to the theoretical bound. Generally, these are either trajectories which were used for training (first or last trials) or ones whose estimated β values corresponded to naive mice (extremely small, consistent with null complexity) or fully trained ones (large β, with values close to the maximal achievable bound of 0).

b. Both value and the complexity cost have to be evaluated from their theoretical expressions and, hence, using the parameters estimated from data. These are estimated by assuming that the behavior is optimal (i.e., minimizes the free energy). Figure 5, however, shows that behavior can lie away from the theoretical curve. Is there a contradiction in the approach, here?

The theoretical curve is an average curve (the control model optimizes the expected value, Eqs. 7 and 11), and therefore the value of individual paths can deviate from it. Estimating the distribution of observed values under the model assumptions is a difficult problem that we did not address. Our approach for estimating the goodness of fit of the model was to use the standard quantifiers of mouse behavior in the water maze (Fig. 3 and related discussion).

c. The grey line that exhibits mean values over mice/trials is presumably obtained from an average over all mice/trials; it displays a non-monotonic evolution of the complexity cost. Since later on, using Fig. 6, the point is made that WT mice show non- monotonicity, but HET mice do not, I wondered why the authors did not distinguish between the two groups also in Fig. 5, which would then have exhibited two different 'average curves'.

We redesigned figure 5 according to your suggestion and split the grey line to two average

curves, one for the WT females and one for the HET females.

8. The authors discuss some of the connections of their work with past work, including on control theory and inference. It may be nice to mention also connections with rate-distortion theory and the information bottleneck.

Done. The main difference between our model and the rate-distortion/information bottleneck framework is that in our case the joint distribution of sensory inputs and optimal motor outputs is unknown. Thus the value functional serves as a proxy for accuracy, or mutual information between the internal representation and the predicted output, and the complexity is a proxy for the compression of the perceptual "signal" by the brain (under the assumption that naive behavior represents maximal compression). In the rate-distortion/information bottleneck case, the joint input-output distribution is generally assumed to be known and the method provides a minimal representation of the input which contains maximal information about the output.

Minor points

9. I suspect that the authors decided to describe the dynamics in a Hamiltonian formalism rather than in a Lagrangian formalism because it yields a Markov property useful, e.g., for discretizing time and for calculating the likelihood of a trajectory in data fitting. It would be good to explain this.

We indeed chose to denote the position and velocity (or momentum) coordinates using the letters p,q based on the convention for generalized coordinates in the Hamiltonian formalism. However, this was not meant to indicate a choice between Hamiltonian or Lagrangian formalisms. Rather, since we already used x to denote the full state vector we simply needed another letter to donate the position component. Since the Newtonian dynamics in our model (linearly) relates position and velocity with their derivatives, i.e., velocities and accelerations, the markov property was obtained simply by using a four dimensional state space which includes velocity as well as position coordinates for defining the state of the mouse.

10. Equation (3): in the upper right block of the matrix, should the elements not be "1/*m*" instead of "1"?

Thanks for pointing this out. We had an inconsistency between Equations 3 and 6 regarding the inclusion of the mass normalization factor, 1/m. Effectively, the former treated p as momentum while the latter as velocity. We now treat p as velocity throughout and specify this in the text.

11. Equation (4):

a. What is the rationale for assuming noise with fixed variance? In principle, a control variable could also be applied to modulate the magnitude of the noise.

Our guiding rationale was to use the simplest model which can provide an adequate description of the behavior. Thus, we made use of the standard Linear Quadratic Gaussian (LQG) formalism which assumes a fixed variance Gaussian noise. An extension of LQG for control dependent noise has been proposed (Todorov, Neural Computation, 2005) and is now mentioned in the discussion of possible extensions of the model.

b. What is the rationale for assuming the presence of noise in both position and momentum?

Assuming separate noise covariances for position and velocity improved the maximum likelihood estimation accuracy of the free model parameters from simulated trajectories compared to a model which assumes noise only the position coordinates.

12. I suggest using square brackets, rather than round brackets, around the argument of functionals (e.g., in Eqs. (7) and (14)), as this notation is more common and is also helpful to distinguish functionals from functions.

Agreed and done.

13. In Eq. (7), the parentheses are missing around the integrand.

Fixed, thank you.
