# Supplementary material for: Value-complexity tradeoff explains mouse navigational learning
Source: PLoS Comput Biol. 2020 Dec 11;16(12):e1008497. doi: 10.1371/journal.pcbi.1008497 (PMC7758052; doi:10.1371/journal.pcbi.1008497)
Supplement: Attachment — Submitted filename: ReviewReport.pdf [file pcbi.1008497.s003.pdf]

Review of the revised manuscript "Value-complexity tradeoff explains mouse navigational learning" by Amir et al.

While authors provide thorough and interesting responses to some of the points raised in the previous reviewer report (points 7-13), responses to other issues raised (points 1-6) are either lacunary or not reflected in the manuscript, or both. I thus find myself in the uncomfortable position of having to comment on a paper which I find quite beautiful and worthy, and whose publication I definitely recommend as it presents a new way of thinking about navigational behavior from an information theoretic and constrained optimization angle—but, at the same time, having to contend with responses by authors who do not seem to take the review process seriously nor wish to include necessary material in the manuscript.

For example:

1) In their response to point 2.a, the authors state that they "added some information at the introduction"—but nothing substantial has been included in the introduction: all I could find in connection with Sanov's theorem is a mention that the complexity cost is "theoretically justified." This will hardly be helpful to the many readers who don't come with a robust mathematical background! The relation of the author's complexity measure to large deviations theory should be presented comprehensively.

2) In response to points 2.b and c, the authors provide a helpful and interesting discussion in their reply, which would indeed be useful to readers of the paper—but it has not been included in the paper in any form, as far as I can tell.

3) In response to point 3.a, no explanation is provided by the authors about the way in which the dynamic programming (Bellman) problem is converted into local differential (or finite difference) equations. If the reader is to be expected to actually understand the approach proposed by the authors, this kind of explanation is necessary! Since the authors claim to be solving an optimization problem, indeed of the Bellman form, the reader should be able to tell why the Ricatti equations fulfill the job—and this even more so in a publication whose readership includes experimentalists. The authors should include a detailed discussion of the mathematics involved here, and of how one converts the Bellman problem into the Ricatti equations.

4) How boundary conditions and transient solutions are treated is an important aspect of the solution of the problem posed by the authors. The question of transient solutions, that arise because of temporal boundary conditions (point 3b), is of particular relevance. In this connection, the authors merely added a brief phrase in the revised manuscript (lines 839-840) that states that transient solutions are neglected. A detailed treatments of boundary conditions and transient solutions should be presented.

5) Figure 4 (point 5) is improved, but some lingering issues remain:
- the low values of beta are not apparent in the illustration (no blue arrow is visible);
- it not clear whether black arrows are closer to red or yellow arrows (both cases appear to occur in the diagram); it would make sense to show also arrows corresponding to the value of beta that best fits the data.

Finally, a minor issue is that, on line 654, Ref [34] is repeated.

In sum, while I find that this paper would be a wonderful addition to the literature, in my view publication would be warranted only if the authors greatly expand the mathematical and technical discussions pertaining to their way of defining and solving the problem, including the points mentioned above.
