# Supplementary material for: Value-complexity tradeoff explains mouse navigational learning
Source: PLoS Comput Biol. 2020 Dec 11;16(12):e1008497. doi: 10.1371/journal.pcbi.1008497 (PMC7758052; doi:10.1371/journal.pcbi.1008497)
Supplement: Attachment — Submitted filename: MWM 2nd revision response letter.pdf [file pcbi.1008497.s004.pdf]

“Value-complexity tradeoff explains mouse navigational learning” - 2nd revision response letter

While authors provide thorough and interesting responses to some of the points raised in the previous reviewer report (points 7-13), responses to other issues raised (points 1-6) are either lacunary or not reflected in the manuscript, or both. I thus find myself in the uncomfortable position of having to comment on a paper which I find quite beautiful and worthy, and whose publication I definitely recommend as it presents a new way of thinking about navigational behavior from an information theoretic and constrained optimization angle—but, at the same time, having to contend with responses by authors who do not seem to take the review process seriously nor wish to include necessary material in the manuscript.
We thank the reviewer again for his or her insightful comments. We added a mathematical appendix providing additional background material concerning the issues raised in points 2a, 3a, 3b. We also addressed, in the main text, the issues raised in points 2b and c, and 5. The main changes to the manuscript are detailed below.

For example:

1) In their response to point 2.a, the authors state that they “added some information at the introduction”—but nothing substantial has been included in the introduction: all I could find in connection with Sanov’s theorem is a mention that the complexity cost is “theoretically justified.” This will hardly be helpful to the many readers who don’t come with a robust mathematical background! The relation of the author’s complexity measure to large deviations theory should be presented comprehensively.
In the section S1 of the mathematical appendix we provide an overview of large deviations theory and justify, using Sanov’s theorem, our use of the Kullback-Liebler divergence between controlled and naive trajectory distributions as a measure of policy complexity.

2) In response to points 2.b and c, the authors provide a helpful and interesting discussion in their reply, which would indeed be useful to readers of the paper—but it has not been included in the paper in any form, as far as I can tell.
We included this discussion regarding the interpretation of the complexity measure in the penultimate paragraph of the introduction.

3) In response to point 3.a, no explanation is provided by the authors about the way in which the dynamic programming (Bellman) problem is converted into local differential (or finite difference) equations. If the reader is to be expected to actually understand the approach proposed by the authors, this kind of explanation is necessary! Since the authors claim to be solving an optimization problem, indeed of the Bellman form, the reader should be able to tell

why the Ricatti equations fulfill the job—and this even more so in a publication whose readership includes experimentalists. The authors should include a detailed discussion of the mathematics involved here, and of how one converts the Bellman problem into the Ricatti equations.
In section S2 of the mathematical appendix we show how the Riccati differential equation is derived from the Hamilton-Jacobi-Belman optimization problem and the associated Euler-Lagrange equations for the corresponding Hamiltonian.

4) How boundary conditions and transient solutions are treated is an important aspect of the solution of the problem posed by the authors. The question of transient solutions, that arise because of temporal boundary conditions (point 3b), is of particular relevance. In this connection, the authors merely added a brief phrase in the revised manuscript (lines 839-840) that states that transient solutions are neglected. A detailed treatments of boundary conditions and transient solutions should be presented.
In section S3 of the mathematical appendix we elaborate upon the issue of temporal boundary conditions and transients. Based on our choice of cost functional structure and parameterization in the inverse optimal control estimation problem (Eq. 38), we demonstrate why the algebraic Riccati equation may be used instead of the differential one for solving the optimal control problem (beta=infinity) in our model. We also rearranged the presentation of the computation of the optimal feedback matrix K (subsection Computing the Optimal Feedback Gain in the Methods section), presenting first the general form of the solution (including temporal transients) and then our approximation using the stationary solution of the algebraic Riccati equation for finite beta values. This approximation is standard in the optimal control literature, based on the rapid (exponential) decay rate of the transient components in the solution of the Riccati differential equation.

5) Figure 4 (point 5) is improved, but some lingering issues remain:
- the low values of beta are not apparent in the illustration (no blue arrow is visible);
- it not clear whether black arrows are closer to red or yellow arrows (both cases appear to occur in the diagram); it would make sense to show also arrows corresponding to the value of beta that best fits the data.
- We adjusted the range of plotted beta values so that all colors are visible.
- We added a dashed black arrow in the insets indicating the velocity vector corresponding to the value of beta that best fits the data.

Finally, a minor issue is that, on line 654, Ref [34] is repeated.
Fixed.

In sum, while I find that this paper would be a wonderful addition to the literature, in my view publication would be warranted only if the authors greatly expand the mathematical and technical discussions pertaining to their way of defining and solving the problem, including the points mentioned above.
